# In memory of Professor Gordon McVie 1945–2021

**DOI:** 10.3332/ecancer.2021.ed110

**Published:** 2021-03-16

**Authors:** Eduardo Cazap

**Affiliations:** Editor in Chief, *e*cancer, 13 King Square Avenue, Bristol, BS2 8HU, UK

**Keywords:** global cancer control, open access, cancer research

## Abstract

The international oncology community is in mourning. Professor Gordon McVie, the co-founder and former Editor in Chief of ecancer, passed away on 20th January 2021. His scientific achievements were broad and demonstrated a curious mind and a tireless commitment to science and cancer care. His achievements in the world of cancer are only surpassed by his kindness, enthusiasm and his love for family and friends.

An international authority on the treatment and research of cancer, Gordon McVie wrote over 350 peer-reviewed articles, editorials and books. Senior Lecturer at the Cancer Research Campaign Oncology Unit, Clinical Research Director at the National Cancer Institute of the Netherlands, Past-President of EORTC, Director General of the Cancer Research Campaign, founding member of the National Cancer Research Institute, UK and Senior Consultant of Clinical Research at the European Institute of Oncology, are only a few of his many relevant positions.

But his favorite contribution was having been the founding editor of the journal ecancermedicalscience. In 2007 Gordon, along with the late Umberto Veronesi, founded a new endeavour, ecancer, a free online global cancer educational platform including the open access journal ecancermedicalscience, which is now supported by a UK charity (the ecancer Global Foundation).

“Forty years in cancer clinical research and people still dying in their thousands. I'd like a better epitaph than that, so I'm still following my curiosity genotype, arranging marriages between lab and clinical scientists, and communicating globally online as founding editor of ecancer.org”

– Professor Gordon McVie via LinkedIn

When conversing with Gordon for the past several years, it became clear to me that his passion was spreading knowledge and global education while overcoming economic and language barriers. My interpretation is that Gordon was evolving from his outstanding scientific and technical expertise to a top-level understanding of cancer in the broader concept of global cancer control.

Luiz Antonio Santini Rodrigues da Silva, former Director General of the National Cancer Institute of Brazil said: “Gordon McVie represented an important impulse for cancer research in INCA BRASIL, supported by the SwissBridge project. He helped us in the design and funding of this project

His dedication to patients was one of his many virtues, as has been recognised by his peers:

Professor Richard Sullivan, Chair of Trustees of the *e*cancer Global Foundation said: Professor Gordon McVie was a wonderful friend, a global pioneer in cancer research and champion of patients.

Many colleagues and friends from around the world have sent their condolences, sharing similar stories about his close relationship with patients, that he was one of the best oncologists of his generation, and an inspiration for cancer researchers and clinical investigators around the world, particularly for reducing the differences in cancer control among rich and developing countries.

Prof Dr Bob Pinedo said “I invited Gordon to work in my newly opened lab at the Netherlands Cancer Institute in 1979. He became particularly interested in our Antimetabolite research to which he made an important contribution. Besides his scientific input, he was a great team player, was very close to his parents and was very proud of his children, often talking about them”.

Gordon McVie will also be remembered with great fondness due to his immense personality and his enormous ability to unite people behind a common goal. On succeeding him as Editor in Chief of *e*cancer, I realised that all the team members had great respect and admiration for him. The international oncology community has lost a global leader, an innovator and a fine researcher but, on top of that, an exceptional friend and gentleman.

